# The Analysis of the Inflorescence miRNome of the Orchid *Orchis italica* Reveals a *DEF*-Like MADS-Box Gene as a New miRNA Target

**DOI:** 10.1371/journal.pone.0097839

**Published:** 2014-05-15

**Authors:** Serena Aceto, Maria Sica, Sofia De Paolo, Valeria D'Argenio, Piergiuseppe Cantiello, Francesco Salvatore, Luciano Gaudio

**Affiliations:** 1 Department of Biology, University of Naples Federico II, Napoli, Italy; 2 CEINGE-Biotecnologie Avanzate, Napoli, Italy; 3 Department of Molecular Medicine and Medical Biotechnologies, University of Naples Federico II, Napoli, Italy; TGen, United States of America

## Abstract

Plant microRNAs (miRNAs) are small, regulatory non-coding RNAs involved in a wide range of biological processes, from organ development to response to stimuli. In recent years, an increasing number of studies on model plant species have highlighted the evolutionary conservation of a high number of miRNA families and the existence of taxon-specific ones. However, few studies have examined miRNAs in non-model species such as orchids, which are characterized by highly diversified floral structures and pollination strategies. Therefore, we analysed a small RNA library of inflorescence tissue of the Mediterranean orchid *Orchis italica* to increase the knowledge on miRNAs in a non-model plant species. The high-throughput sequencing and analysis of a small RNA library of inflorescence of *O. italica* revealed 23 conserved and 161 putative novel miRNA families. Among the putative miRNA targets, experimental validation demonstrated that a *DEF*-like MADS-box transcript is cleaved by the homolog of miR5179 of *O. italica*. The presence of conserved miRNA families in the inflorescence of *O. italica* indicates that the basic developmental flower regulatory mechanisms mediated by miRNAs are maintained through evolution. Because, according to the “orchid code” theory, *DEF*-like genes exert a key function in the diversification of tepals and lip, the cleavage-mediated inhibitory activity of miR5179 on a *OitaDEF*-like transcript suggests that, in orchids, miRNAs play an important role in the diversification of the perianth organs.

## Introduction

MicroRNAs (miRNAs) are small (18–25 nt) non-coding RNAs that regulate gene expression at post-transcriptional level. They originate from single-stranded precursors (pre-miRNAs) able to self-pair and form hairpin structures. In plants, the Dicer1-like RNAse III enzyme excises the miRNA/miRNA* duplex from the pre-miRNA. Subsequently, the guide strand of the duplex is recruited by an RNA-induced silencing complex (RISC), whose core component is the protein ARGONAUTE1 (AGO1). This complex permits the interaction between the miRNA and its target mRNA, thereby regulating its expression through mRNA cleavage, translational repression or epigenetic modifications [1], [2]. Plant miRNAs play crucial roles in different processes, from leaf and root morphogenesis to floral induction, organ formation, reproduction and stress response [2], [3], [4], [5].

Advances in next-generation sequencing techniques have prompted a plethora of studies on miRNAs in both model and non-model species and have led to the development of specific *in silico* analysis tools [6], [7], [8], [9]. Recently, the high-throughput sequencing approach has been applied to the study of miRNAs in two orchid species, *Phalaenopsis aphrodite* and *Erycina pusilla*, both belonging to the sub-family Epidendroideae (Orchidaceae) [10], [11]. Conserved and novel miRNAs have been identified in both species, and some differences between the two species have been detected.

The Orchidaceae family is one of the largest among flowering plants and is characterized by highly specialized reproductive strategies and extremely diversified flowers. Despite their remarkable morphological diversity, orchid flowers share bilateral symmetry and the organization into three outer tepals, two inner lateral tepals and an inner median tepal (labellum or lip). In addition, the column, the inner whorl, is constituted by male and female reproductive tissues fused together. At the base of the column is located the ovary, whose maturation is triggered by pollination. The pollen grains (pollinia) are located at the top of the column.

The (A)BCDE model describes the function of different functional classes of homeotic genes involved in flower development [12], [13], [14]. All the genes involved in the (A)BCDE model, except the class A gene *APETALA2* (*AP2*), are MADS-box transcriptional factors. Although this model is applicable to most flowering plants, in orchids it shows modifications in the expression domains of the class B *GLOBOSA*-like (*GLO*-like) and *DEFICIENS*-like (*DEF*-like) genes [15], [16], [17], [18] and of the class C (*AGAMOUS*-like) and D (*SEEDSTICK*-like) genes [19], [20]. To date, the only gene belonging to the (A)BCDE model and negatively regulated by a miRNA is *AP2*, whose transcript is cleaved by miR172 with a conserved mechanism present also in orchids [10], [21].

The aim of this study is to characterize the miRNA population expressed in the floral buds of *Orchis italica*, a wild Mediterranean orchid species belonging to the sub-family Orchidoideae. In the attempt to shed light on conserved and novel miRNAs expressed in a non-model plant species, we performed a computational analysis of the floral miRNAs of *O. italica* based on deep sequencing data in order to identify possible new miRNA targets involved in floral development.

## Materials and Methods

### Deep sequencing of small RNAs of *O. italica*

Total RNA was extracted from inflorescence of *O. italica* before anthesis (∼9 mm diameter size) using the Trizol Reagent (Ambion). Ten different florets from a single inflorescence were used as starting material. Although not synchronous, the selected florets were collected from the bottom of the inflorescence, displayed approximately the same size and could be considered in the same developmental stage. After DNase treatment, RNA was quantified using the Nanodrop 2000 c spectrophotometer (ThermoScientific). The quality of the extracted RNA was checked using the Agilent 2100 BioAnalyzer (Agilent). Small RNA library preparation and sequencing were carried out according to the manufacturer instruction (Illumina). The library obtained was sequenced using the MiSeq instrument (Illumina). The raw reads were deposited at NCBI under the accession PRJNA222705.

### *In silico* analysis of the library

Raw reads of inflorescence tissue of *O. italica* were processed using the plant version of the UEA sRNA workbench [22], [23] to remove the adaptor sequence, the low quality reads and the reads with abundance lower than 5. Filtering options were set to exclude reads shorter than 18 and longer than 35 nucleotides. After removal of tRNA and rRNA contaminants, reads were collapsed and the read count for identical sequences was summed.

Using the filtered dataset of *O. italica* as query, standalone BLAST was conducted against the known plant mature and hairpin miRNA sequences downloaded from mirBase 20 [24] to search for conserved miRNAs in the inflorescence of *O. italica*. In addition, short reads were blasted against the miRNAs expressed in the floral bud of the orchid *P. aphrodite* [10]. Only reads matching at least 18 nt and with less than 3 mismatches were considered positive.

We used the miRDeep-P software [25], which is specific for plant miRNA analysis, to search for new small RNA sequences in the short reads of inflorescence tissue of *O. italica* and matching the criteria of plant putative miRNAs. The first step of this analysis is based on the bowtie alignment of the cleaned and collapsed short reads to a reference transcriptome. Unfortunately, the *O. italica* transcriptome is not available and only a few orchid transcriptomes [10], [26], [27], [28] are deposited in public databases as Sequence Read Archive files. As more data are available for the orchid *P. aphrodite* than for the other orchids (including tissue-specific sRNA libraries), we used this species as reference although it belongs to the distantly-related sub-family Epidendroideae. We assembled the transcriptome of *P. aphrodite* starting from the deep sequencing files deposited in the Sequence Read Archive under accession code SRA030409. After adapter removal, filtering and collapsing, reads of vegetative, seed and inflorescence tissue of *P. aphrodite* were separately assembled using the Trinity software [29]. The three assembled tissue-specific transcriptomes were compared to obtain a not-redundant collection of unigenes that was used as reference transcriptome. The annotation of the assembled transcriptome was performed using the FastAnnotator online tool [30]. Under miRDeep-P, the bowtie alignment was performed allowing for a maximum of three mismatches and reads mapping to multiple positions (maximum 15) were retained. The potential miRNA precursors were then selected by setting the maximum length to 250 nt, and their secondary structure was predicted using RNAfold.

### Putative target analysis

We used the psRNATarget online tool [31] for the prediction of the putative targets of the short reads matching the plant miRNA criteria. The cleaned short reads of *O. italica* were analyzed against the newly assembled transcriptome of *P. aphrodite* setting the search parameters to the default value (maximum expectation 3.0).

Using more stringent search parameters (maximum expectation 0.0), the cleaned reads were analyzed also against different transcripts of genes involved in flower development of *O. italica* that we have previously isolated (*OrcLFY* [GenBank: AB088851], *OitaAP2* [GenBank: KF152921], *OrcPI* [GenBank: AB094985], *OrcPI2* [GenBank: AB537504], *OitaAG* [GenBank: JX205496], *OitaSTK* [GenBank: JX205497]). Within these transcripts, the class B MADS-box genes are represented by *OrcPI* and *OrcPI2*, both of which are members of the GLO-like lineage. We decided to isolate also the other class B genes of *O. italica* belonging to the DEF-like lineage and to verify if they are targets of miRNAs.

One microgram of total RNA extracted from floral buds of *O. italica* was reverse transcribed using the Advantage RT-PCR kit (Clontech) and an oligo dT primer. The MADS-box degenerate primer MADS_F (Table 1) and a poly-T primer were used to amplify 1 µl of first strand cDNA using the LongAmp Taq PCR Kit (New England Biolabs) [19], [32]. The amplification products were cloned into the pGEM-T Easy vector (Promega); more than 50 clones were sequenced using the plasmid primers T7 and SP6 and sequencing reactions were run on an ABI 310 Automated Sequencer (Applied Biosystems).

**Table 1:** Sequence of the primers used.

Name	Sequence (5′-3′)	Locus	Putative miRNA target
MADS_F	AAGATAGAGAATCCDACDAACD	MADS-box	
OitaDEF1F	CCTTCGCAGGGAGATAAGGCAAAGGA	*OitaDEF1*	
OitaDEF2F	CCTTCGGAAGGAGATAAGGCAGAGGA	*OitaDEF2*	
OitaDEF3F	CCTGAGGAGGGAGATAAGGCAGAGAA	*OitaDEF3*	
OitaDEF4F	TCTGAGGAGGGATGTAAGACAGAGGA	*OitaDEF4*	
OitaDEF1R1	TCATGCATAAGGGCCCTGTATACTTC	*OitaDEF1*	
OitaDEF2R1	TCATGCACTAGGGCCATGCACATTTC	*OitaDEF2*	
OitaDEF3R1	TCACGGAGTAAGCTCTTGTGGGTTTC	*OitaDEF3*	
OitaDEF4R1	TCACGCAGCAAATTATGGTGTGTCTC	*OitaDEF4*	
OitaDEF1R2	GTAAGTGTCTGTTTGCGTGGCGATCA	*OitaDEF1*	
OitaDEF2R2	GTAAGTGTCAGTTTGGGTAGCGATCA	*OitaDEF2*	
OitaDEF3R2	GTATGTATCAGTCTGGGTGCTAATGC	*OitaDEF3*	
OitaDEF4R2	ATAGGTGTCTGTCTGCGTACTGATTA	*OitaDEF4*	
OitaActF	TCGCGACCTCACCAATGTAC	*OitaAct*	
OitaActR	CCGCTGTAGTTGTGAATGAATAGC	*OitaAct*	
IN_3340	TCTCGGACCAGGCTTCATTCC	miR166	Leucine-zipper transcription factor
IN_36629	TCGCTTGGTGCAGGTCGGGA	miR168	AGO1
IN_33620	TTCCACAGCTTTCTTGAACTG	miR396	Growth regulating factor 4
IN_16410	TCGATAAACCTCTGCATCCGG	miR162	Dicer1-like
IN_32138	AAGCTCAGGAGGGATAGCGCC	miR390	TAS3
IN_30974	CAGCCAAGGATGACTTGCCGA	miR169	NF-YA
IN_33680	TGCCTGGCTCCCTGTATGCCA	miR160	Auxine response factor
IN_26041	TTTTGCTCAAGACCGCGCAAC	miR5179	*DEF*-like genes
IN_20892	ATATGAGCTCAAATCTAAGCTTG	Unknown miRNA	Leucine-rich repeat protein kinase
IN_27201	AAACTCTCTGAAATCACCCGAGAGG	Unknown miRNA	Transmembrane kinase
5.8S	ACGTCCGCCTGGGCGTCAAGC	Ribosomal 5.8S	

Sequence of the primers used to isolate the *DEF*-like cDNAs, to analyze their tissue expression and the expression of the selected conserved and novel miRNAs of *O. italica*. Relative to the selected miRNAs, the putative targets are listed (best score).

BLAST search of the obtained sequences revealed four different *DEF*-like cDNAs that were deposited as *OitaDEF1-4* [DDBJ: AB857726, AB857727, AB857728, AB857729, respectively]. Virtual translation of the coding sequence of the *OitaDEF*-like genes was aligned to other DEF-like and GLO-like (class B MADS-box proteins) sequences present in GenBank using ClustalW. The Neighbor Joining (NJ) tree of the resulting alignment was constructed using the MEGA5 software [33], performing 1000 bootstrap replicates.

Based on the predicted position of the putative miRNA target site on the *OitaDEF*-like cDNAs, specific reverse primers were designed and used to verify the presence of the cleavage product performing a modified 5′-RACE experiment [34]. All the *DEF*-like specific reverse primers annealed downstream of the predicted putative miRNA cleavage site. Using the RLM-RACE GeneRace kit (Invitrogen), the 5′ adaptor was ligated to the 5′-terminus of the RNA extracted from inflorescence tissue of *O. italica* (500 ng) without any enzymatic treatment to remove the 5′ cap. After reverse transcription, cDNA was amplified using the GeneRace 5′ Primer and the OitaDEF1R1, OitaDEF2R1, OitaDEF3R1 or the OitaDEF4R1 specific reverse primers (Table 1). A second PCR reaction was conducted on 1 µl of the first reaction using the nested adaptor forward primer and the nested OitaDEF1R2, OitaDEF2R2, OitaDEF3R2 or the OitaDEF4R2 specific reverse primers (Table 1). The amplification products were cloned and sequenced as described above.

### Tissue expression analysis

Using the protocol described above, we extracted total RNA from leaf, outer and inner tepal, lip and column dissected from the inflorescence of *O. italica* before anthesis. In this bud stage, the flower organs are already formed and cell division is completed, while cell elongation is still ongoing. We extracted total RNA from ovary tissue before and after manual fertilization (3, 7 and 10 days after pollination). Within the immature ovules of *O. italica* (before pollination) the megaspore mother cell is undergoing the first meiotic division. At 3 days after pollination, the female gametophyte has completed its development; at 7 days after pollination, fertilization has occurred and the seeds are in the early developmental stage; at 10 days after pollination the seeds are mature (Barone Lumaga, personal communication).

Within the miRNAs in the inflorescence of *O. italica*, we selected 10 of those most highly expressed and/or with interesting putative targets (e.g. involved in miRNA biogenesis, in flower development, etc), and analyzed their relative expression profile in the different tissues. Eight of the selected miRNAs correspond to known plant miRNAs and two fulfil the plant miRNA criteria but do not correspond to any known miRNAs. The primer sequences of the selected miRNAs are listed in Table 1.

The Poly(T) Adaptor RT-PCR method [35] was used to analyse the expression pattern of the selected miRNAs starting from 350 ng of total RNA from each tissue. A poly-T adaptor was ligated to the 3′-terminus of the miRNAs and subsequently reverse transcribed. The forward primer specific for each selected miRNA was used in combination with the poly-T adaptor reverse primer during the Real Time PCR experiments. The latter were performed on 30 ng of the first strand cDNA from each tissue using the 5.8S RNA as endogenous control gene [35] and the SYBR Green PCR Master Mix (Life Technologies). Reactions were conducted in technical and biological triplicates. The Real Time PCR Miner online tool [36] was used to calculate PCR efficiency (E) and optimal threshold cycle (C_T_) for each well. The mean relative expression ratio (Rn) and standard deviation of the selected miRNAs in the different tissues was calculated using the 5.8S RNA as the endogenous control gene with the following formula Rn = (1+E_miRNA_)^-CTmiRNA^/(1+E_5.8S_)^-CT 5.8S^. The Rn values were multiplied by an arbitrary value (100,000). Differences in relative expression levels of the 10 selected miRNAs in the various tissues were assessed by ANOVA followed by the Tukey HSD post hoc test. Real Time PCR product of several samples was cloned and sequenced to exclude the presence of amplification artefacts.

Real Time-PCR experiments were conducted to analyze the relative expression of the *OitaDEF*-like genes of *O. italica* in the same tissues. Total RNA (350 ng) from each tissue was reverse transcribed as described above. The Real Time PCR reactions were conducted using 30 ng of the first strand cDNA from each tissue and the following primer pairs: OitaDEF1F/OitaDEF1R1, OitaDEF2F/OitaDEF2R1, OitaDEF3F/OitaDEF3R1, OitaDEF4F/OitaDEF4R1 (Table 1). The actin *OitaAct* gene was used as endogenous control [GenBank: AB630020]. Amplification conditions are reported elsewhere [16]. The amplification data were processed as described above.

## Results and Discussion

### Conserved and novel miRNAs in the inflorescence of *O. italica*

**Figure 1:**
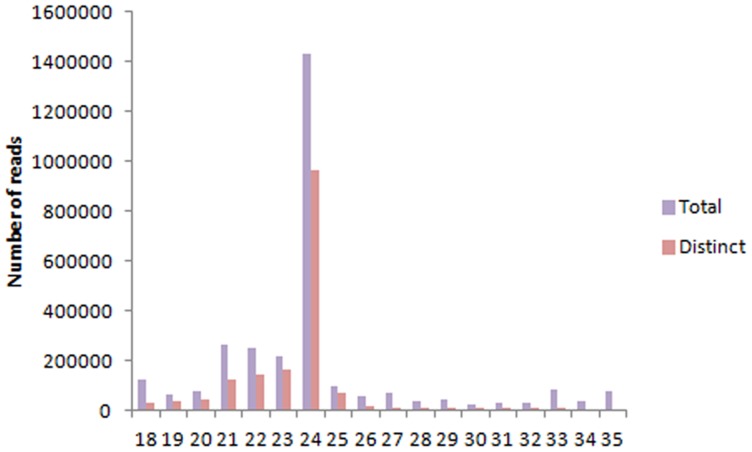
Length distribution of total and distinct short reads in the inflorescence of *O. italica*.

**Figure 2:**
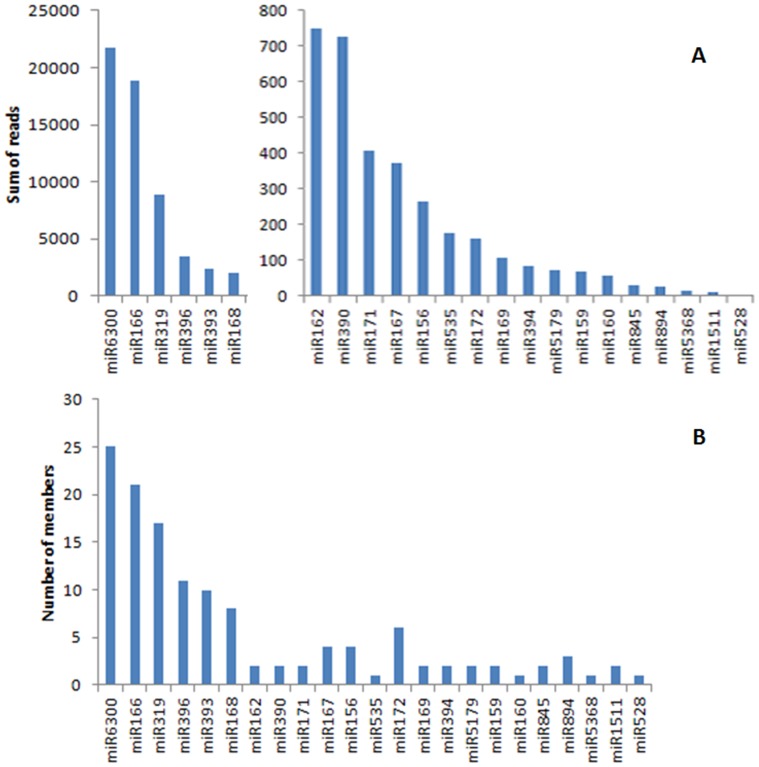
Abundance of conserved miRNAs in the inflorescence of *O. italica*. **A) Sum of reads; B) number of members.**

The high-throughput sequencing of the small RNA library of inflorescence tissue of *O. italica* produced 4,718,127 total reads. Adaptor trimming and length filtering (18–35 nucleotides) reduced the total reads to 3,019,126. Short sequences representing less than 5 reads, and tRNA and rRNA contaminant sequences were removed thereby resulting in 1,064,237 total and 37,818 distinct reads (Table 2, File S1). Within the specified length range, the number of reads was highest between 21–24 nucleotides, with a peak at 24 nt (Figure 1). This is in agreement with the distribution pattern of small RNAs in other plant species such as *Arabidopsis thaliana* [37], *Caragana intermedia* [8], *Solanum tuberosum* [38], *Avicennia marina* [7], *Oryza sativa* [39] and the orchid *P. aphrodite* [10]. BLAST analysis revealed 175 distinct sequences in the inflorescence tissue that corresponded to 23 known plant miRNAs and 6 star sequences (Figure 2A, Table 3, File S2). Among the former, the most abundant are miR6300, miR166, miR319, miR396, miR393 and miR168, which are also the most heterogeneous, being represented by the highest number of members (Figure 2B).

**Table 2:** Summary statistics of small RNA sequencing in inflorescence of *O. italica*.

	Total	Distinct
Raw reads	4,718,127	2,100,557
Remaining after adaptor removal	4,412,197	1,953,969
Remaining after length range filtering (18–35)	3,019,126	1,672,887
Remaining after low-complexity filtering	3,018,960	1,672,730
Remaining after minimum abundance filtering (5)	1,161,336	40,054
Remaining after invalid sequence filtering	1,160,925	40,027
Remaining after tRNA/rRNA filtering	1,064,237	37,818

**Table 3:** Comparison between miRNA and miRNA* detected in inflorescence of *O. italica*.

miRNA family	Sum of reads		Number of members	
	miRNA	miRNA*	miRNA	miRNA*
miR166	18,796	35,338	21	21
miR162	750	18	2	2
miR171	406	511	2	13
miR156	263	22	4	3
miR159	69	631	2	4
miR164	0	107	0	3

The comparison of the conserved miRNAs between the inflorescence tissue of *O. italica* and *P. aphrodite* (File S3) revealed that almost all miRNAs are shared in both species, albeit with some differences in their relative abundance. For example, miR166 and miR168 are among the most abundant miRNAs in the floral buds of both *O. italica* and *P. aphrodite*, miR319 and miR396 are expressed at a higher level in *O. italica* than in *Phalaenopsis*, miR528 is highly expressed in *Phalaenopsis* whereas it has only five reads in *O. italica*. In addition, miR6300 is highly expressed in *O. italica* whereas it is absent from *Phalaenopsis*. These differences could reflect the different developmental stage of the flower bud used to construct the small RNA libraries in the two orchid species and the size difference of the two libraries [10].

**Figure 3:**
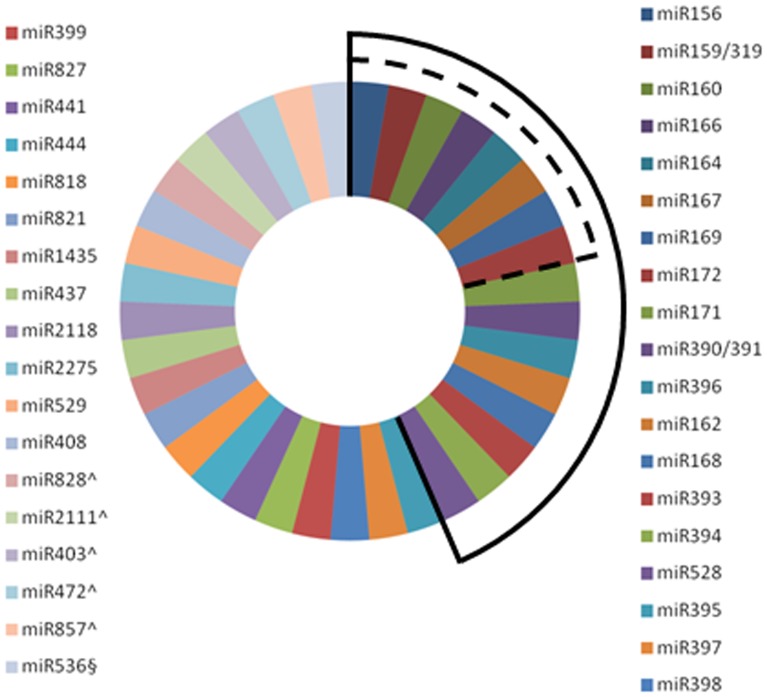
Evolutionary conserved miRNAs detected in the inflorescence of *O. italica*. The list of the evolutionary conserved miRNAs is from Cuperus et al. 2011 [40]. Dotted lines group together the evolutionary conserved miRNAs involved in flower development as described in Luo et al. 2013 [41]. Continuous lines group together the evolutionary conserved miRNAs detected in the inflorescence of *O. italica*. ∧ indicates miRNA specific of dicots; § indicates miRNA specific of Bryophyta and Lycopodiophyta.

The known miRNAs identified in the inflorescence of *O. italica* correspond to 16 of the 37 evolutionary and taxonomically conserved miRNA families [40] (Figure 3). Given that 5 of these conserved miRNAs are specific of dicots and 1 is specific of Bryophyta and Lycopodiophyta, approximately 52% (16/31) of the evolutionary conserved miRNAs is expressed in the inflorescence of *O. italica*. Among them, one (miR528) is specific of monocots. In addition, all the 8 evolutionary conserved miRNA families involved in flower development [41] are present in the inflorescence of *O. italica* (Figure 3), even though miR164 is present only as star sequence (miR164*). These results suggest that the basic regulatory pathways of flower development mediated by miRNAs are maintained through evolution. In addition to mature miRNAs, we detected 6 miRNAs* (currently annotated in miRBase as -3p) in the inflorescence of *O. italica* (Table 3). They have almost the same number of members as their respective mature miRNA, although they are differently accumulated. In particular, there are more miR166*, miR159* and miR171* reads than miR166, miR159 and miR171 reads, respectively, whereas the other miRNAs* have fewer reads than their mature miRNAs. In addition, miR164 is present only as star sequence. These differences in the abundance of mature and star miRNAs might be due to a functional role of miRNA* when the number of reads is high, or to miRNA* degradation due to its exclusion from the silencing complex in favour of the mature miRNA [6], [42], [43].

Using the newly assembled transcriptome of *P. aphrodite* as reference, we looked for novel miRNAs using miRDeep-P, and identified 478 distinct sequences of inflorescence that matched the criteria of plant miRNA structures. Among these, 109 correspond to known plant miRNA families present in miRBase and to miRNA sequences of *P. aphrodite*, and 100 show similarity with known miRNAs although limited to 11–15 nucleotides. The latter putative miRNAs are not abundant (number of reads ranging from 5 to 154) and might represent known miRNAs belonging to families with variable nucleotide sequences. We analysed the remaining 296 distinct reads using the CD-HIT suite [44], and identified 161 clusters with a sequence identity cut-off of 80%. With the exception of 3 sequences of 26, 28 and 31 nt, respectively, their distribution length ranges between 19–24 nt, with a peak at 21 nt (File S3). These putative novel miRNAs should be considered orchid-specific as their prediction was made using the *P. aphrodite* transcriptome as reference.

### Putative targets

**Figure 4:**
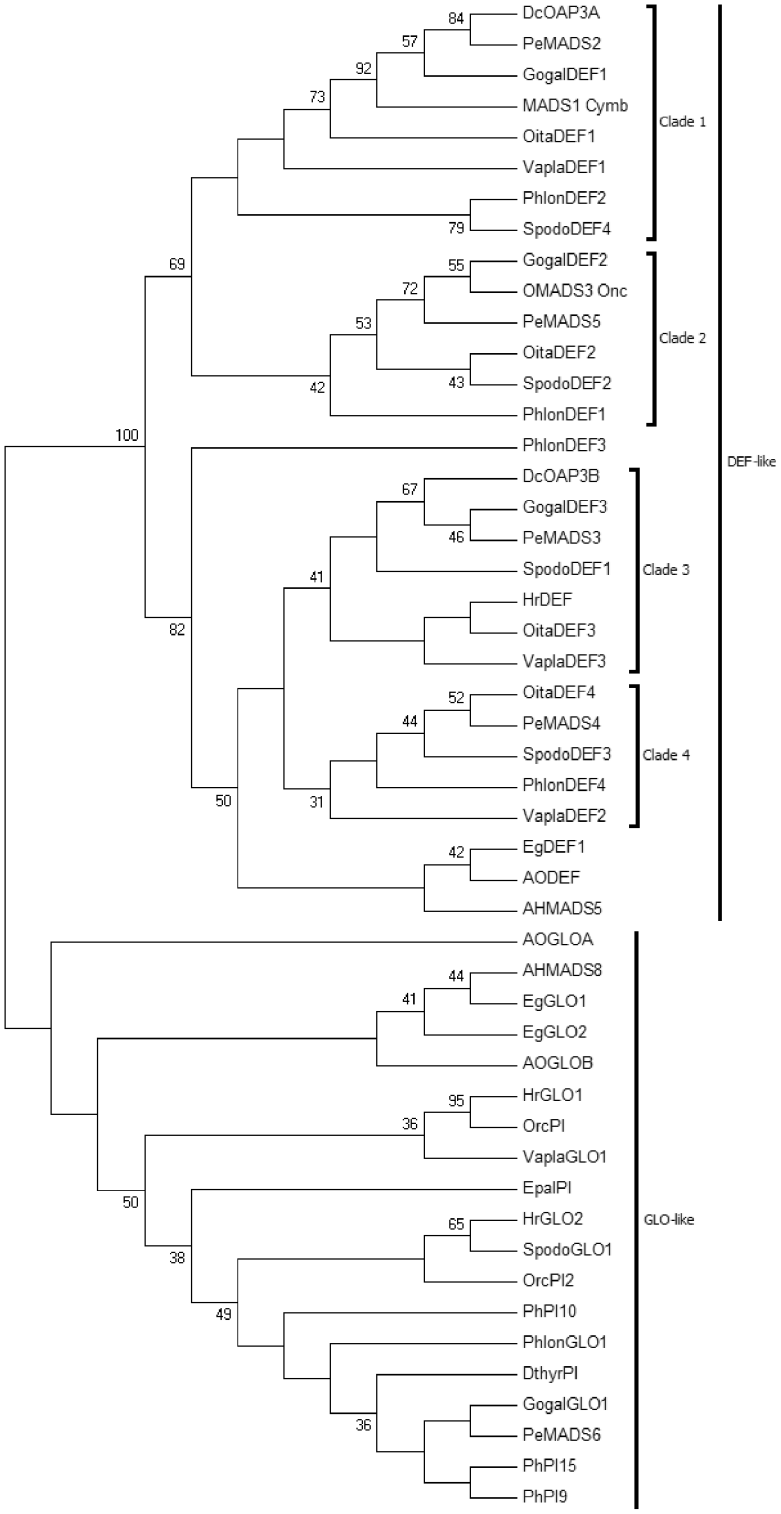
NJ bootstrap consensus tree of the examined amino acid sequences of the DEF-like and GLO-like genes. Numbers represent the bootstrap percentages. Taxon labels reflect the GenBank annotation and do not always correspond to the clade name of the orchid DEF-like lineage. *Alpinia hainanensis* AHMADS8 [AAT99429], AHMADS5 [AAT99427];*Asparagus officinalis* AODEF [BAC75969], AOGLOA [BAD13495], AOGLOB [BAD13496]; *Cymbidium hybrid cultivar* MADS1 Cymb [ABG78568]; *Dendrobium crumenatum* DcOAP3A [AAZ95248], DcOAP3B [AAZ95249]; *Dendrobium thyrsiflorum* DthyrPI [AAY86363]; *Elaeis guineensis* EgGLO1 [AAQ13915], EgGLO2 [AAQ03229], EgDEF1 [AAW66883]; *Epipactis palustris* EpalPI [AAY56590]; *Gongora galeata* GogalDEF1 [ACR16036], GogalDEF2 [ACR16037], GogalDEF3 [ACR16038], GogalGLO1 [ACR16039]; *Habenaria radiata* HrDEF [BAH03320], HrGLO1 [BAH03322], HrGLO2 [BAH03321]; *Oncidium hybrid cultivar* OMADS3 Onc [AAO45824]; *Orchis italica* OitaDEF1 [BAO00916], OitaDEF2 [BAO00917http://getentry.ddbj.nig.ac.jp/getentry/dad/BAO00916.1/?filetype=html], OitaDEF3 [BAO00918], OitaDEF4 [BAO00919], OrcPI [BAC22579], OrcPI2 [BAI78360]; *Phalaenopsis equestris* PeMADS2 [AAR26628], PeMADS3 [AAR26629], PeMADS4 [AAR26626], PeMADS5 [AAR26627], PeMADS6 [AAV28175]; *Phalaenopsis hybrid cultivar* PhPI9 [AAV28175], PhPI10 [AAV28490], PhPI15 [AAV28491]; *Phragmipedium longifolium* PhlonDEF1 [ACR16044], PhlonDEF2 [ACR16045], PhlonDEF3 [ACR16046], PhlonDEF4 [ACR16047], PhlonGLO1 [ACR16048]; *Spiranthes odorata* SpodoDEF1 [ACR16049], SpodoDEF2 [ACR16050], SpodoDEF3 [ACR16051], SpodoDEF4 [ACR16052], SpodoGLO1 [ACR16053]; *Vanilla planifolia* VaplaDEF1 [ACR16054], VaplaDEF2 [ACR16055], VaplaDEF3 [ACR16056], VaplaGLO1 [ACR16057].

Using a MADS-box degenerated primer and a poly-T primer, we identified four different cDNAs belonging to the *DEF*-like lineage of the class B MADS-box gene family. These cDNAs range from the first codon (the ATG start codon) to the poly-A tail. *OitaDEF1* is 804 bp with an ORF of 681 bp; *OitaDEF2* is 893 bp with an ORF of 603 bp; *OitaDEF3* is 733 bp with an ORF of 672 bp; *OitaDEF4* is 818 bp with and ORF of 675 bp. The NJ tree constructed on the amino acid alignment of the sequence of the virtual translation of these cDNAs with other DEF-like and GLO-like proteins present in GenBank shows that they belong to the known orchid DEF-like clades 1–4 (Figure 4). The tree topology confirms that the DEF-like genes duplications predate the origin of the Orchidaceae [45]. All the OitaDEF-like putative proteins have the typical domain organization of the MIKC transcription factors: the highly conserved MADS-box domain, the moderately conserved intervening I-domain and keratin-like K-domain and the variable C-terminal domain [46].

The putative targets of the known and novel miRNAs identified in our study were predicted using the psRNATarget online tool. A total of 5349 putative targets were detected against the transcriptome of *P. aphrodite* using the default settings of psRNATarget (maximum expectation 3.0). These putative targets correspond to 1456 distinct Gene Ontology (GO) annotations involved in a broad variety of biological processes including cellular and metabolic processes, regulatory processes, developmental processes, response to stimuli, etc. Homologs of known miRNA targets were predicted for several conserved miRNAs of *O. italica*, including *AP2* (target of miR172), *Dicer1-like* (target of miR162), *MYB* (target of miR159/319), *NF-YA* (target of miR169), *HD-ZIP* transcription factors (targets of miR166), etc.

**Figure 5:**
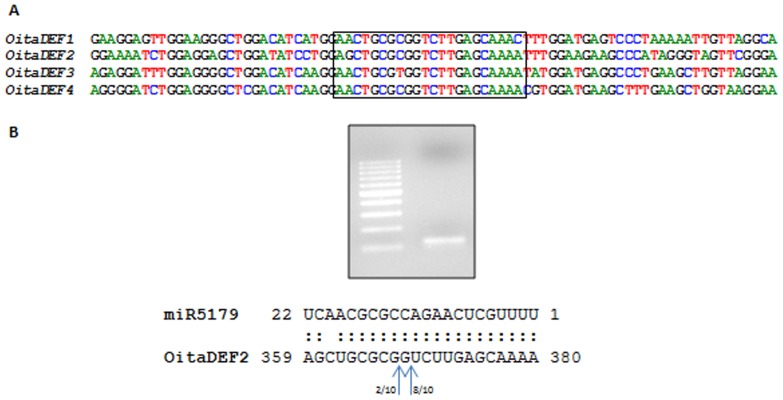
Cleavage of the homolog of miR5179 on *OitaDEF2* in *O. italica*. A) Nucleotide alignment of the putative target site of miR5179 on *OitaDEF2* and its surrounding region with the corresponding region on *OitaDEF1*, *OitaDEF3* and *OitaDEF4*. The nucleotide sequences span from the position 330 to 410, considering the first nucleotide of the ATG start codon as position 1. B) Agarose gel electrophoresis of the modified 5′ RACE experiment on *OitaDEF2* run together with the 100 bp ladder (Fermentas). The alignment of the miR5179 and its target site on *OitaDEF4* is reported and the arrows indicate the position of the cleavage and the number of clones corresponding to each site as deduced by the cloning and sequencing of the obtained fragment (see Methods).

When the psRNATarget analysis was conducted against the coding transcripts of *O. italica*, we used a more stringent parameter setting (maximum expectation 0.0), obtaining positive results for *OitaAP2* and the two class B MADS-box transcripts *OitaDEF2* and *OitaDEF4*. As expected, the *O. italica* miRNAs that target the *OitaAP2* transcript are homologs of miR172 [47]. The two *O. italica* miRNAs INF_28502 and INF_26041 are predicted to target the *OitaDEF2* and *OitaDEF4* transcripts in a corresponding position. Both these miRNAs are homologs of miR5179 of *Oryza sativa* and *Brachypodium distachyon* whose function is unknown. Recently, an orchid-specific miRNA that putatively cleaves the transcripts of class B MADS-box genes was identified in *E. pusilla* and *P. aphrodite*, without any further characterization [11]. Using a modified 5′ RACE protocol, we successfully amplified a fragment of the expected size (129 bp) and sequence representing the cleavage product of miR5179 on the Oita*DEF2* transcript of *O. italica* (Figure 5), thus confirming that this class B MADS-box gene is a target of the homolog of miR5179 in *O. italica*. Although the *in silico* analysis predicted also *OitaDEF4* as a possible target of the homolog of miR5179 with the same expectation value of *OitaDEF2*, we did not detected any cleavage product of this miRNA on *OitaDEF4*. In addition, even though the nucleotide sequence of the putative target site of miR5179 is quite conserved also in the *OitaDEF1* and *OitaDEF3* transcripts (Figure 5A), the respective cleavage product was not detectable. This result could be related to the nucleotide differences of the regions surrounding the predicted target site on the four *DEF*-like transcripts, which results in decreased accessibility of the RISC complex on *OitaDEF4* as well as on *OitaDEF1* and *OitaDEF3*. Alternatively, it is conceivable that the activity of miR5179 on the transcript of *OitaDEF1*, *OitaDEF3* and *OitaDEF4* might be mediated by translational repression or epigenetic modifications, without a direct cleavage.

### Tissue expression

**Figure 6:**
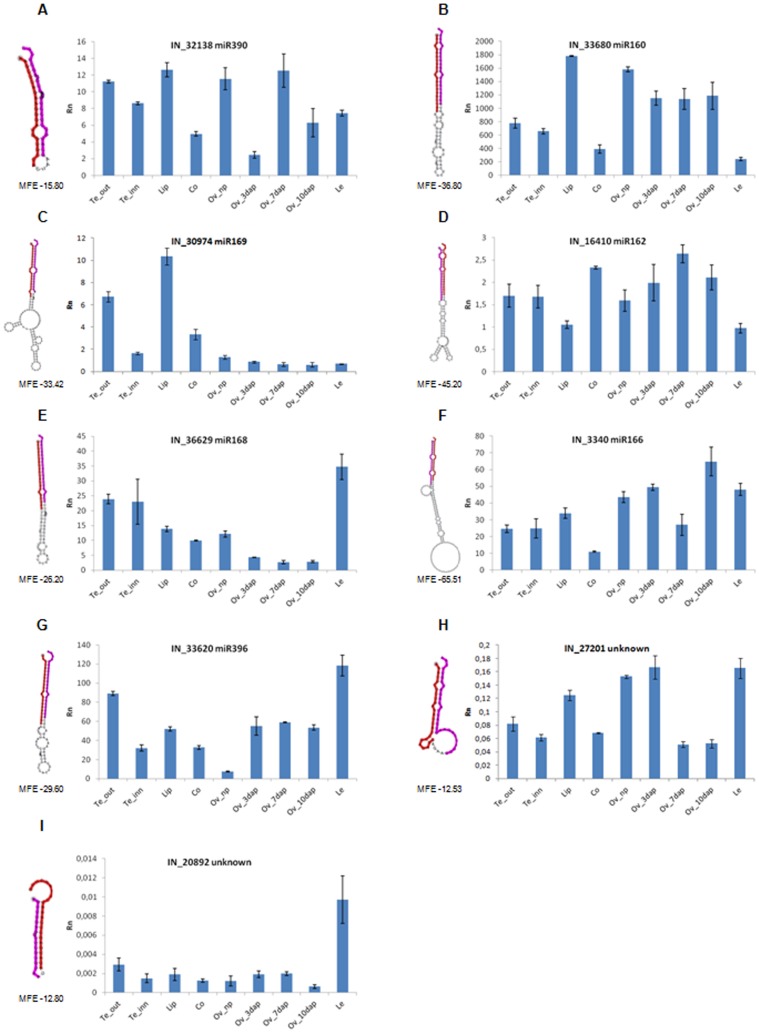
Relative expression pattern of selected conserved and putative novel miRNAs in different tissues of *O. italica*. The predicted structure of the pre-miRNA is reported on the left of each graph, where the miRNA and miRNA* sequences are shown in red and pink, respectively. MFE, minimum free energy; Rn, relative expression ratio; Te_out, outer tepal; Te_inn, inner tepal; Co, column; Ov_np, not pollinated ovary; Ov_3dap, Ov_7dap, Ov_10dap, ovary 3, 7 and 10 days after pollination, respectively; Le, leaf. Bars indicate the standard deviation.

**Figure 7:**
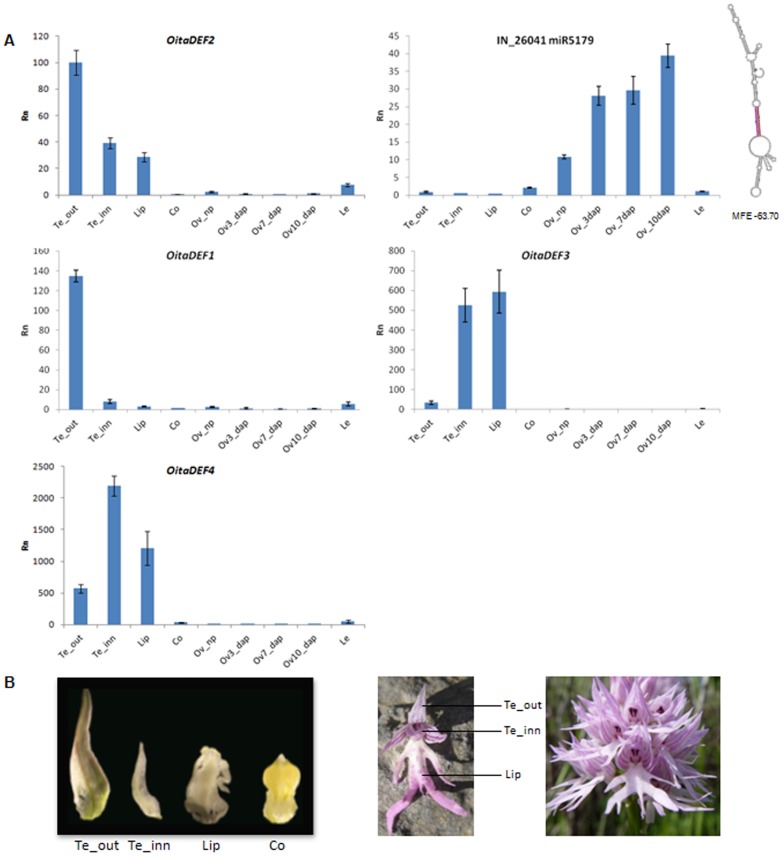
Relative expression pattern of miR5179 and of the *OitaDEF*-like genes of *O. italica*. A) Expression profile of the *OitaDEF1-4* genes and of the homolog of miR5179 in different tissues of *O. italica*. The predicted structure of the miR5179 pre-miRNA of *O. italica* is reported at the rigth side of the expression graph, where the miRNA and miRNA* sequences are shown in red and pink, respectively. MFE, minimum free energy; Rn, relative expression ratio; Te_out, outer tepal; Te_inn, inner tepal; Co, column; Ov_np, not pollinated ovary; Ov_3dap, Ov_7dap, Ov_10dap, ovary 3, 7 and 10 days after pollination, respectively; Le, leaf. Bars indicate the standard deviation. B) From left to right: floral tissues of *O. italica* used in the expression analysis; single floret and inflorescence of *O. italica* after anthesis.

Ten miRNAs of *O. italica*, 8 matching known plant miRNAs and 2 putative novel (Table 1), were selected to conduct a relative expression analysis within different inflorescence tissues (Figures 6 and 7). As the Real Time PCR experiments were conducted on the different floral whorls, while the raw reads count was performed on the whole inflorescence, differences were detected between the two datasets. The examined miRNAs belong to families with a different number of members (Figure 2B) and the primers used in the Real Time PCR experiments amplify only part of them. This can further explain the differences between the two datasets.

The expression pattern of the homolog of miR390 in *O. italica* (Figure 6A) reveals a not uniform expression profile in the examined tissues. miR390 triggers the biogenesis of the *trans*-acting silencing RNAs (ta-siRNAs) by directly interacting with the *TAS3* mRNA [48]. These ta-siRNAs specifically inhibit the auxine response factor mRNAs *ARF2*, *ARF3* and *ARF4*, which are involved in the responses to the phytohormone auxin through a pathway that is highly conserved among land plants [48].

The miR160 homolog is highly expressed in all floral organs of *O. italica* (Figure 6B), particularly in the lip and ovary, which is in agreement with its role in the shaping of the inflorescence and of the floral organs, as well as in fertility [49], [50].

As shown in Figure 6C, the expression of the homolog of miR169 of *O. italica* is higher in the tepals and lip than in the column, ovary or leaf. The targets of miR169 are *NF-YA* transcription factors that activate the expression of the class C MADS-box gene *AGAMOUS* (*AG*) [51]. Consequently, miR169 restricts, in an indirect manner, the expression domain of the *AG* gene to the inner floral whorls. The expression pattern of miR169 in *O. italica* is complementary to that reported for *OitaAG* in the same floral tissues [19], which demonstrates that the role of miR169 is highly conserved among plants.

The expression of the homolog of miR162 of *O. italica* is relatively low in all the examined tissues, particularly in the lip and leaf (Figure 6D). miR162 targets the transcript of the *Dicer1-like* gene that is involved in the biogenesis of the miRNAs [52]. The low levels of miR162 could reflect a high level of processing of the pre-miRNAs within the floral tissues of *O. italica*.

The expression of the homolog of miR168 of *O. italica* is higher in tepals, lip, column, not pollinated ovary and leaf than in ovary after pollination (Figure 6E). miR168 cleaves the transcripts of the *AGO1* gene that encodes a core component of the RISC complex [53]. In addition, miR168 seems to be involved in stress responses and signal transduction [54].

The homolog of miR166 of *O. italica* was expressed at variable levels within the examined tissues, with the lowest value detected in the column and the highest in ovary 10 days after pollination (Figure 6F). miR166 targets the mRNA of the HD-ZIP III transcription factors and is involved in shoot apical and lateral meristem formation, organ polarity, and vascular development [55], [56].

The homolog of miR396 of *O. italica* is expressed at various levels in the floral and leaf tissue; it is low in the ovary before pollination and high in outer tepals and leaf (Figure 6G). miR396 is involved in flower and leaf development targeting the transcripts of growth regulating factors [57].

The expression profile of two putative novel orchid-specific miRNAs is shown in Figure 6H and I. The predicted putative targets of IN_27201 and IN_20892 are a transmembrane kinase and a leucine-rich repeat protein kinase, respectively (Table 1). The expression of both the putative novel miRNAs was lower than that of the selected conserved miRNAs. This is in agreement with reports that the expression of the non-conserved miRNAs is lower compared with that of conserved miRNAs [7], [8].

Finally, as shown in Figure 7A, the miR5179 of *O. italica* is expressed at low levels in the organs of the perianth. Its expression is considerably higher in ovary tissue than in the other examined tissues, and reaches its maximum level at 10 days after pollination. This pattern is opposite to that of miR5179's putative target transcript *OitaDEF2*, which is highly expressed in outer tepals, less expressed in inner tepals and lip, whereas its expression is very low in the column and ovary. This complementary expression profile fully fits with the possible repressive role of miR5179 on *OitaDEF2*. Figure 7A also shows the expression profile of the other *DEF*-like genes of *O. italica*. *OitaDEF1* is expressed mainly in outer tepals, whereas the expression of *OitaDEF3* and *OitaDEF4* is higher in inner tepals and lip than in the other tissues.

According to the “orchid code” theory, the class B MADS-box genes belonging to the DEF-like group are responsible for the morphological differences among the organs of the orchid perianth (outer and inner tepals and lip) (Figure 7B) [18], [58]. Fine regulation of the relative expression levels of the four *DEF*-like genes differentiates the tepals (in which the clade 1 and 2 genes play a predominant role) from the lip (in which the clade 3 and 4 genes play a predominant role) [18]. The expression pattern of the *DEF*-like genes in *O. italica* is in agreement with the “orchid code” theory, and supports the concept that the co-expression and the relative abundance of the four *DEF*-like genes (not simply their presence/absence) is responsible for the morphological diversification of the orchid perianth. In addition, our results suggest that a miRNA regulates the activity of a clade 2 *DEF*-like gene, which implicates miR5179 in the differentiation of the organs of the perianth in orchids.

## Conclusions

The analysis of the inflorescence miRNome of the orchid *O. italica* revealed the presence of conserved and novel miRNAs. The *in silico* search for the possible miRNA targets showed a conserved miRNA cleavage site within the four *OitaDEF*-like transcripts, which we experimentally validated for *OitaDEF2*. This result suggests that miRNAs play an important role in the diversification of the organs of the perianth in orchids through the inhibitory regulation of the clade 2 *DEF*-like gene, and highlights the potential of next-generation sequencing in the study of non model plant species. Different mechanisms might act to regulate the expression level of the other *DEF*-like genes, suggesting the existence of lineage-specific regulatory mechanisms contributing to the functional specialization of the *DEF*-like clades in orchids.

## Supporting Information

File S1 **Distinct reads of the small RNA library of inflorescence of** ***O. italica*** **after pre-processing.** The file is in fasta format and for each sequence the number of reads is indicated after “_x”.
(TXT)

File S2 **Results of the BLAST analysis of the cleaned reads of** ***O. italica*** **versus the plant miRNAs annotated in miRBase20.**
(XLSX)

File S3 **Results of the BLAST analysis of the cleaned reads of** ***O. italica*** **versus the miRNAs of floral buds of** ***P. aphrodite*** **.**
(XLSX)

File S4 **Length distribution of the clustered orchid-specific putative miRNAs in the inflorescence of** ***O. italica*** **.**
(TIF)
