# Supplementary figures and images for: The Analysis of the Inflorescence miRNome of the Orchid Orchis italica Reveals a DEF-Like MADS-Box Gene as a New miRNA Target
Source: PLoS One. 2014 May 15;9(5):e97839. doi: 10.1371/journal.pone.0097839 (PMC4022656; doi:10.1371/journal.pone.0097839)

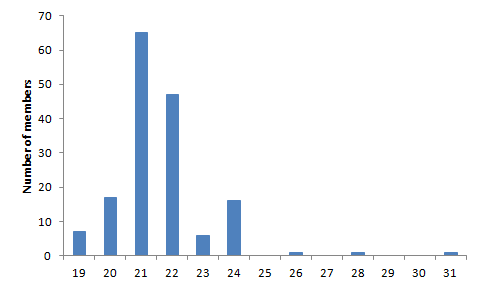

Supplement: File S4 — Length distribution of the clustered orchid-specific putative miRNAs in the inflorescence of O. italica . (TIF) [file pone.0097839.s004.tif]
